# The Progestin-Only Contraceptive Medroxyprogesterone Acetate, but Not Norethisterone Acetate, Enhances HIV-1 Vpr-Mediated Apoptosis in Human CD4^+^ T Cells through the Glucocorticoid Receptor

**DOI:** 10.1371/journal.pone.0062895

**Published:** 2013-05-03

**Authors:** Michele Tomasicchio, Chanel Avenant, Andrea Du Toit, Roslyn M. Ray, Janet P. Hapgood

**Affiliations:** Department of Molecular and Cell Biology, University of Cape Town, Cape Town, Western Province, South Africa; St. Jude Children's Research Hospital, United States of America

## Abstract

The glucocorticoid receptor (GR) regulates several physiological functions, including immune function and apoptosis. The HIV-1 virus accessory protein, viral protein R (Vpr), can modulate the transcriptional response of the GR. Glucocorticoids (GCs) and Vpr have been reported to induce apoptosis in various cells, including T-cells. We have previously shown that the injectable contraceptive, medroxyprogesterone acetate (MPA) is a partial to full agonist for the GR, unlike norethisterone acetate (NET-A). We investigated the functional cross talk between the GR and Vpr in inducing apoptosis in CD4^+^ T-cells, in the absence and presence of GCs and these progestins, as well as progesterone. By using flow cytometry, we show that, in contrast to NET-A and progesterone, the synthetic GR ligand dexamethasone (Dex), cortisol and MPA induce apoptosis in primary CD4^+^ T-cells. Furthermore, the C-terminal part of the Vpr peptide, or HIV-1 pseudovirus, together with Dex or MPA further increased the apoptotic phenotype, unlike NET-A and progesterone. By a combination of Western blotting, PCR and the use of receptor- selective agonists, we provide evidence that the GR and the estrogen receptor are the only steroid receptors expressed in peripheral blood mononuclear cells. These results, together with the findings that RU486, a GR antagonist, prevents Dex-, MPA- and Vpr-mediated apoptosis, provide evidence for the first time that GR agonists or partial agonists increase apoptosis in primary CD4^+^ T-cells via the GR. We show that apoptotic induction involves differential expression of key apoptotic genes by both Vpr and GCs/MPA. This work suggests that contraceptive doses of MPA but not NET-A or physiological doses of progesterone could potentially accelerate depletion of CD4^+^ T-cells in a GR-dependent fashion in HIV-1 positive women, thereby contributing to immunodeficiency. The results imply that choice of progestin used in contraception may be critical to susceptibility and progression of diseases such as HIV-1.

## Introduction

Globally women account for ∼49% of HIV infections [1], with greater prevalence among young women vulnerable to both pregnancy and HIV-infection than in men [2]. In Sub-Saharan Africa, 59% of all those infected are women [3]. There is substantial evidence from clinical studies that hormonal contraception increases HIV-1 acquisition and transmission in young women and disease progression, although some of the findings are controversial, and some studies report no significant effects [4]–[15]. Of particular interest are the relative effects of the two most commonly used injectable contraceptives, MPA and norethisterone enanthate (NET-EN), in HIV-1 infection and AIDS progression. MPA, administered for contraception as Depo-MPA (DMPA) or Depo-Provera, is a 150 mg three-monthly intramuscular injection that is used by millions of women worldwide and is widely used in Sub-Saharan Africa and other areas with high HIV-1 prevalence [6], [14]–[17]. NET-EN is a 200 mg two-monthly injectable that is used less than MPA, although in countries like South Africa, its usage varies and is high in some regions [8], [17]–[19]. Both contraceptives have been shown to be highly effective and relatively safe regarding most risk factors investigated [20]. However, there is evidence that DMPA but not NET-EN increases HIV infectivity [5], [6], [8], [10], [15], [21], [22]. Increases in both HIV-1 and HSV shedding have been reported in women using contraception [23]–[25], as well as the presence of more viral variants and higher viral loads in HIV-1 infected DMPA users than non-users [7], consistent with an increase in HIV-1 transmission found for DMPA users [5]. DMPA usage has also been associated with increased acquisition of cervical chlamydial and gonococcal infections [26]. In addition, while there is evidence both for and against an increase in disease progression in the absence of antiretroviral drugs (ARVs) for HIV-1 positive DMPA users [7], [9], [11]–[13], to our knowledge no information is available for effects of NET-EN. Whether DMPA or NET-EN affect disease progression in HIV-1 positive antiretroviral users remains to be established, although one study suggests no significant change in CD4^+^ counts for DMPA users with and without antiretroviral drug usage [27]. Adjusted hazard ratios (HRs), (reflecting the fold increased risk relative to no contraception), of between 1.5 and 4.5 fold have been recently reported for DMPA (author’s response in Gray [4], [5], [8], [10]), while one study reported an HR as high as 10.4 for DMPA [28]. However, establishing indisputable evidence from such clinical observational studies is extremely difficult due to multiple confounding factors such as the degree of exposure to HIV-1, condom usage, HSV-2 exposure as well as varying ages of women that have been enrolled in these studies [15]. Therefore, a central question remains as to what extent and via which mechanisms different synthetic progestins affect HIV-1 pathogenesis at contraceptive doses and at various target sites, a question perhaps best answered by *ex vivo* studies.

At the cellular level, progestins mediate their effects via alterations in transcription of specific genes in target cells by binding to and regulating the activity of steroid receptors, which are ligand-activated transcription factors [29], [30]. Although progestins are designed to act like the natural ligand progesterone (P4) via the progesterone receptor (PR), they are likely to exert very different off target side-effects due to their differential affinities and activities via other members of the steroid receptor family of receptors [29]–[31]. NET-EN is converted to norethisterone (NET) in the body, while water soluble derivatives of NET-EN such as NET or norethisterone acetate (NET-A are used orally for hormone replacement therapy) [27], [28]. We have shown that MPA and NET-A have different affinities for and activities via the glucocorticoid receptor (GR) [32]–[34]. MPA binds to the GR with a relatively high affinity and acts as a full to partial agonist for the GR, whereas NET-A and P4 bind to the GR with about 100-fold lower affinity and have little or no activity via the GR. This differential activity via the GR suggest that MPA and NET may exert different effects on HIV-1 pathogenesis via the GR, in particular different effects on immune function, since the GR regulates transcription of a wide variety of genes involved in inflammation, immunity, and apoptosis [35], [36]. Several different mechanisms could contribute to the observed effects of DMPA usage on HIV-1 pathogenesis, including alterations in the composition of mucosal microflora and thinning of the cervical/vaginal epithelium. However, the high affinity of MPA for the GR and the known effects of the GR on immune function suggest that effects of DMPA on both systemic and local immunity via the GR may be highly significant.

Although very few studies have investigated this hypothesis, some do show that MPA affects immune function *in vivo* in animals and humans. Two studies in mice provide evidence that MPA suppresses immune function to increase susceptibility to infections or reduce defense against disease in mice, at similar doses to those of women using DMPA [37], [38]. Studies in primates have shown that DMPA reduces systemic immune responses in SIV-infected macaques [39], [40]. MPA used at high doses in cancer therapy is known to cause significant systemic immunosuppression in patients [41]–[44] and a decrease in T-cell numbers and proliferation in breast cancer patients [41], [44]. Furthermore, DMPA as a contraceptive has been shown to compromise cell-mediated immune status [45] and causes increased recruitment of inflammatory cells in cervical vaginal lavages in women [46]. A recent *ex vivo* study in primary immune function and cervical cells from patients showed that MPA, unlike P4, suppresses both innate and adaptive immune mechanisms at concentrations within the range of peak serum concentrations found in DMPA users [47], [48]. Importantly, the findings from the group of Hel [47] showing significant repression of IFNλ in peripheral blood mononuclear cells (PBMCs) and lavages from DMPA users but not non-contraceptive users strongly supports the idea that DMPA concentrations *in vivo* are sufficient for immunosuppression. Interestingly, P4 is also known to regulate both the innate and adaptive immune response in the female reproductive tract, other mucosal tissues as well as systemic immune function in humans. However, the precise mechanisms and receptors involved in this regulation are not well understood, but appear to be specific for different target sites and cell types [6], [49], [50]. In contrast, very little is known about the effects of NET-EN/NET/NET-A on immune function in women.

HIV-1 infection is characterized by rapid and extensive CD4^+^ T-cell depletion and eventual immunodeficiency. HIV-1-induced apoptosis appears to play an important role in depletion of CD4^+^ T-cells, decreasing immune responses to infection and facilitating viral persistence and increased viral loads and transmission rates [51]. Furthermore, the loss of CD4^+^ T-cells correlates with disease progression and increases in opportunistic infections [52]. Although the exact mechanisms and role of apoptosis during disease progression remain to be resolved, several HIV-1 proteins have been implicated in inducing apoptosis in T-cells, including the 96 amino acid HIV-1 accessory protein viral protein R (Vpr) [53]. Besides apoptosis, Vpr has been implicated to play a part in other cellular functions such as cell cycle arrest at G2/M phase and transport of the pre-initiation complex [53]. Vpr is packaged within the virus particle where it is thought to be involved in the early stages of viral replication through transactivation of the HIV-1 long terminal repeat (LTR) [53]. The clinical observations that mutations in key Vpr residues are associated with normal capacity to replicate but loss of cytotoxicity [54] and long-term non-progressive HIV-1 infections [55], support an important cytotoxic role for Vpr in HIV-1 infection. Such a cytotoxic role may be exerted by both virus-associated as well as virus-free Vpr, since functional Vpr protein has been purified from serum and cerebrospinal fluid of infected patients [56]–[58]. Vpr in the plasma of HIV-1 infected individuals is present at similar concentrations as the p24 antigen and has the ability to self-penetrate cells (transduction properties) and to elicit its effects, including apoptosis, in non-infected bystander cells [56], [59]. Mapping experiments of the Vpr protein indicated that amino acids 1–52 are important for the transduction properties of Vpr, but not for induction of apoptosis [60]. The C-terminus (amino acids 52–96) of Vpr, in particular amino acids 71–82 (71-HFRIGCRHSRI-82), have been shown to be indispensable for apoptotic function [61]–[64]. Although Vpr can induce apoptosis via the extrinsic pathway in neuronal and epithelial cells [65]–[68], it has been implicated to act predominately through the intrinsic pathway in a number of other cell lines and primary cells, including T-cells [69].

Reminiscent of the effects of Vpr on apoptosis, GCs, like cortisol, (F) acting via the GR, are also potent inducers of apoptosis in a number of different cells, including T-cells [35]. Several lines of evidence suggest that Vpr regulates transcription of host and viral genes via the GR [70]. The mechanism may involve an interaction of Vpr with the GR to modulate GR transcriptional activity, as Vpr has been reported to associate with the GR *in vitro* and modulate transcription of both host and viral genes [71], [72]. This interaction was reported to occur via a signature LXXLL steroid receptor co-activator motif [73]. Consistent with a role for the GR in mediating Vpr effects on apoptosis at the transcriptional level, it has been shown that RU486, a GR antagonist, prevents Vpr-mediated apoptosis in the Jurkat T-cell line [74].

Cross talk between Vpr and the GR raises the question as to how different GR ligands such as MPA would affect GR- and Vpr-mediated T-cell apoptosis. As both the GR and Vpr have been implicated to play a role in apoptosis in a number of cell lines and primary cells, we sought to investigate the possible cross talk between the GR and Vpr in modulating apoptosis in the presence of the progestins, MPA and NET-A, and P4 in PMBCs.

## Materials and Methods

### Ethics Statement

Anonymous buffy packs, otherwise normally discarded, were obtained from the Western Province Blood Transfusion (WPBT) services in Pinelands, Cape Town. Written informed consent was obtained from donors by WPBT and records kept by WPBT. The Ethics Committee of the University of Cape Town (N05/11/187) approved the procedure (SFREC_ 04_2010).

### Plasmids, Western Blotting and Antibodies

The plasmids used in this study were as follows: pcDNA3-hGR (GR) plasmid was a gift from Prof. D.W. Ray (Centre for Molecular Medicine, School of Clinical and Laboratory Sciences, Faculty of Medical and Human Sciences, University of Manchester, UK). pMT-PR-B (PR) was obtained from Prof. S. Okret (Karolinka Institute, Sweden). pRS-hMR (MR) expression plasmid was obtained from Prof. R.M. Evans (University of California, USA). pSV-hAR (AR) was a kind gift from Frank Claessens (Catholic University of Leuven, Belguim). pSG5-hER (ER) was obtained from F. Gannon (EMBL, Germany). The positive controls for each steroid receptor were generated in COS-1 cells (ATCC). Briefly, COS-1 cells were seeded at a density of 1×10^5^ cells in a 12-well plate. After 24 hrs the cells were transfected with 1 µg of the steroid receptor expression vector using X-tremeGENE 9 DNA Transfection Reagent (Roche Applied Sciences) according to the manufacturer’s specifications. The next day whole cell lysates were prepared using a N-[Tris(hydroxymethyl)methyl]-3-aminopropanesulfonic acid (TAPS) buffer (0.1 M TAPS, pH 9.5) on ice as described by Ronacher *et al* [34]. PBMC lysates were also prepared in TAPS buffer from approximately 4×10^6^ cells.

Western blotting was performed essentially as previously described [75]. All antibodies were purchased from Santa Cruz biotechnology (USA, California). Antibodies included anti-androgen receptor (AR, C-441, sc-7305) anti-estrogen receptor (ER, MC-20, sc-542), anti-GR (H-300, sc-8992), anti-mineralocorticoid receptor (MR, C-19, sc-6861), anti-PR (C-20, sc-539) and anti-glyceraldehyde-3-phosphate dehydrogenase (GAPDH, sc-47724).

### Conventional PCR

Conventional PCR was performed using GoTaq DNA polymerase (Promega, USA, M3001) with the steroid receptor specific primers (Table 1) according to the manufacturer’s specifications. Initial denaturation was for 90 sec at 95°C, while final extension was for 5 mins at 72°C. The cycling parameters for 35 cycles are shown in Table 1.

**Table 1:** Primers used for conventional PCR.

Target Gene	Primer Sequence	Cycling Parameters	Product size (bp)
AR	F: 5′-CAGGAAAGCGACTTCACCGCACC-3′R: 5′-ATCAGGCAGGTCTTCTGGGGTGG-3′	95°C (45 sec), 60°C (45 sec), 72°C (45 sec)	209
ER alpha	F: 5′- TCGACGCCAGGGTGGCAGAGR: 5′-TGGTGCACTGGTTGGTGGCTGG-3′	95°C (45 sec), 60°C (45 sec), 72°C (45 sec)	218
GR	F: 5′-TGCTGTGTTTTGCTCCTGATCTG-3′R: 5′-TGTCAGTTGATAAAACCGCTGCC-3′	95°C (45 sec), 53°C (45 sec), 72°C (45 sec)	299
MR	F: 5′-GAGCAGTGGAAGGGCAACAC-3′R: 5′-TGGCTGCTCCTCGTGAATCC-3′	95°C (45 sec), 60°C (45 sec), 72°C (45 sec)	182
PR A and PR B	F: 5′-GTGCTCAAGGAGGGCCTGCCG-3′R: 5′-TGTGCTGCCCTTCCATTGCCC-3′	95°C (45 sec), 60°C (45 sec), 72°C (45 sec)	214

F = Forward; R = Reverse.

### PBMC Isolation, Cell Culture and Test Compounds

Buffy packs were obtained from healthy donors who were negative for HIV, syphilis and hepatitis B and C. PBMCs were isolated using Histopaque (H1077 Hybri-Max™; Sigma-Aldrich, South Africa) density centrifugation with Leucosep tubes (Greiner Bio-One, Germany) according to the manufacturer’s instructions [76]. PBMCs were cultured in high glucose (4.5 g/ml) Roswell Park Memorial Institute medium (RPMI) (Gibco, South Africa) supplemented with 10% (*v/v*) charcoal-stripped fetal calf serum (c-s FCS) (Highveld Biological, South Africa), 2 mM L-glutamine (Sigma-Aldrich, South Africa), 0.1 mg/mL sodium pyruvate (Sigma-Aldrich, South Africa), 100 IU/ml penicillin and 100 µg/ml streptomycin (Sigma-Aldrich, South Africa) at 37°C in a water jacket incubator (90% humidity and 5% CO_2_). Note that each figure shows the results of experiments using PBMCs isolated from different donors. Dex ((11β,16α)-9-fluoro-11,17,21-trihydroxy-16-methylpregna-1,4-diene-3,20-dione), MPA (Medroxyprogesterone 17-acetate), NET-A (19-Norethindrone acetate), NET (19-Norethindrone), P4 (progesterone), R5020 (17,21-dimethyl-19-norpregna-4,9-dien-3,20-dione), Mib (mibolerone), E2 (estradiol), Ald (Aldosterone) and RU486 (Mifepristone) were purchased from Sigma-Aldrich, South Africa.

### Cell Treatment without Vpr Peptide and Flow Cytrometric Detection of Apoptosis

Approximately 1×10^6^ PBMCs/ml RPMI were seeded into a 5 mL Becton-Dickinson Falcon tube (352063). Cells were then treated with either Dex, F, MPA, NET-A, P4 or vehicle control (EtOH) at the concentrations indicated in the figure legends for 24 hrs at 37°C. After treatment, cells were surface stained and the apoptotic phenotype detected using the annexin V PE apoptosis detection kit I according to the manufacturer’s specifications (Becton-Dickinson-Biosciences; 559763). The following antibodies were used to discriminate between cellular populations in PBMCs: 1 µL anti-CD4 fluorescein isothiocyanate (FITC) (Becton-Dickinson, 555346), 2 µL anti-CD14 allophycocyanin (APC) (Becton-Dickinson, 555399) and 1 µL anti-CD3 APC (Becton-Dickinson, 555342) in 50 µL PBS. Samples were acquired using a Becton-Dickinson FACS Calibur flow cytometer and analysed using Flo Jo software (Treestar, Inc, Ashland, Ore).

### Cell Treatment with Vpr Peptide

The C-terminal Vpr peptide (including amino acids 52–96; GNTWAGVEAIIRILQQLLFIHFRIGCRHSRIGVTRGRRARNGASRS) was a kind gift from Dr Jeffrey Kopp (NIDDK, National Institutes of Health, Bethesda, USA). Freshly isolated PBMCs were treated with 5 µM Vpr peptide as previously described [61]. Briefly, approximately ten million PBMCs were re-suspended in 1 mL of balanced isotonic glucose-HEPES buffer (2.4% glucose, 13 mM HEPES, 68 mM NaCl, 1.3 mM KCl, 4 mM Na_2_HP0_4_ and 0.7 mM KH_2_PO_4_, pH 7.2). One million cells (100 µL) were treated with 2.5 µg (5 µM) Vpr peptide for 30 min at 37°C. The cells were washed and then cultured in RPMI. A bovine serum albumin (BSA) trypic digest was prepared according to a protocol obtained from the Sanford-Burnham Medical Research Institute (Lo Jolla California, USA [77]) and served as a control peptide wherever Vpr peptide was not added. Control peptide was added at a final equivalent concentration as Vpr, in mass/volume i.e. 25 mg/mL. Cells were treated with the test compounds as indicated in the figure legends for 24 hrs at 37°C and apoptosis was detected as indicated above. Note that we found that incubation with this buffer resulted in an increase in basal apoptosis of the CD4^+^ T-cell population from about 2.5% to about 5% (data not shown), which masked apoptotic effects with MPA alone.

### HIV-1 Pseudovirus Generation and Infection

HIV-1 pseudovirus was generated as described by Jochmann *et al* [78]. HEK293T cells (obtained from ATCC) were seeded at a density of 8×10^5^ cells/well in a 6-well plate in high glucose (1 g/ml) phenol red-containing Dulbecco’s Modified Eagles Medium (DMEM) (Sigma-Aldrich) supplemented with 10% (*v/v*) fetal calf serum (FCS) (Highveld Biological, South Africa), 0.1 mg/mL sodium pyruvate (Sigma, South Africa), 100 IU/ml penicillin and 100 µg/ml streptomycin (Gibco Invitrogen) at 37°C in a water jacket incubator (90% humidity and 5% CO_2_). The next day the cells were washed with PBS and phenol red free DMEM (supplemented as described above) was added to each well. Cells were then transfected with 5 µg pSG3.1 (containing the HIV-1 genomic sequences with a mutated envelope (env) gene. (AIDS Research and Reference Reagent Program; [79]) and pDU15A (encoding the HIV-1 envelope) [80] using X-tremeGENE 9 DNA transfection reagent (Roche, South Africa) according to the manufacturer’s specifications. Cells were incubated for 3 days at 37°C, the medium passed through a 0.22 µM filter and charcoal-striped (cs) FCS (Highveld Biological, South Africa) was added to a final concentration of 40%. The viral stocks were aliquotted and stored at −80°C until use. The titre of the pseudotyped viruses was determined using the Reed Muench method and expressed as log TCID_50_/ml [81]. Prior to infection, PBMCs were activated with 5 µg/ml phytohemagglutinin (PHA) (Sigma Aldrich, South Africa) and 20 U/ml recombinant human interleukin-2 (rhIL-2) (Roche,South Africa) for 3 days as previously described [82]. For pseudovirus infection, pseudovirus was added to obtain a multiplicity of infection (MOI) of 0.00005 and incubated for 3 days before stimulation and flow cytometric detection of apoptosis. A standard p24 assay (Aalto Bio Reagents Ltd, Dublin Ireland) was used to confirm that the cells were infected.

### RNA Isolation, cDNA Synthesis and Real Time PCR

Approximately twenty million PBMCs were treated with 5 µM Vpr or control peptide in 1 mL of balanced isotonic glucose-HEPES buffer as described previously. Cells were then treated in the presence or absence of 100 nM Dex, MPA, NET-A or P4 for 24 hrs. The cells were harvested by centrifugation at 350×*g* and RNA was extracted using Tri Reagent (Sigma-Aldrich, South Africa) according to manufacturer’s instructions. RNA was reverse transcribed with oligo-dT priming, using the Transcriptor High Fidelity cDNA Synthesis Kit (Roche, South Africa), and an equal volume of each cDNA synthesis reaction was used as template for real time PCR, using the Sensimix dT Kit (Quantace, London). Quantitative PCR was carried out using primers for Bim and Bcl-2 (Table 2). GAPDH was used as a housekeeping gene for normalization (Table 2; [83]). Initial denaturation and final extension was as for conventional PCR while the cycling parameters for 40 cycles are shown in Table 2. Standard curves were used to determine the efficiency of each primer set, and the relative expression of the genes of interest in each sample was calculated according to the Pfaffl mathematical model [84].

**Table 2:** Primers used for real time PCR.

Target Gene	Primer Sequence	Cycling Parameters	Product size (bp)
GAPDH	F: 5′-TGAACGGGAAGCTCACTGG-3′R: 5′-TCCACCACCCTGTTGCTGTA-3′	95°C (10 sec), 55°C (10 sec), 72°C (10 sec)	307
Bcl-2	F: 5′-TTGTGGCCTTCTTTGAGTTCGGTG-3′R: 5′-GTACAGTTCCACAAAGGCATCCCA-3′	95°C (10 sec), 60°C (10 sec), 72°C (10 sec)	167
Bim	F:5′-GAGTGTGACCGAGAAGGTAGACAATTGC-3′R: 5′-CCTTCACCTCCGTGATTGCCTTC-3′	95°C (10 sec), 55°C (10 sec), 72°C (10 sec)	125

F = Forward; R = Reverse.

### Statistical Analysis

All experiments were performed with PBMCs isolated from at least 3 different donors and at least two independent experiments were performed. All data was normalized to appropriate controls. Data were analysed for statistical significance by One-way ANOVA and appropriate post-tests as indicated in the figure legends using GraphPad Prism software. *, **, and *** indicate p<0.05, p<0.01 and p<0.005, respectively. For dose response analysis, a non-parametric statistical trend test was performed across the concentration range for each compound, using the Wilcox rank-sum test, as further extended by Cuzick [85].

## Results

An important question is whether doses of DMPA and NET-EN used for injectable contraception, and physiological concentrations of endogenous P4, are sufficient to cause significant effects on immune function via the GR *in vivo*. MPA concentrations in the serum of DMPA users are reported to be in the range of 4.5 to 65 nM a few days after injection of 150 mg and then to average at about 2.6 nM for about three months [48], [86]–[89]. NET has been shown to reach a peak plasma concentration of 50 nM a few days after intramuscular injection of NET-EN, followed by an average concentration of about 13 nM for about four months [90]–[92]. The concentration of endogenous P4 in the serum of premenopausal women varies from 0.65 nM to about 80 nM between the follicular and luteal phases, respectively, while reaching about 600 nM during pregnancy [29]. MPA has a high relative binding affinity for the GR (K_i_ of 10.8 nM), similar to that of F (10–20 nM) [93], whereas NET-A and P4 have lower affinity for the GR (K_i_ of 270 and 215 nM respectively) [34], [94], [95]. In order to investigate effects of P4 and progestins via the GR on apoptosis, experiments were thus performed at single concentrations required for near or full saturation of the GR (10–50×K_d_ or K_i_), as well as by dose response analysis using doses spanning the range of concentrations found in the serum of DMPA and NET-EN users, or doses spanning physiologically relevant concentrations for P4. Since NET-EN is not soluble in aqueous solution, we used the water soluble derivatives NET-A or NET [29].

### GCs and the Progestin MPA, but not NET-A or P4, Induce Apoptosis in CD4^+^ T-cells

**Figure 1:**
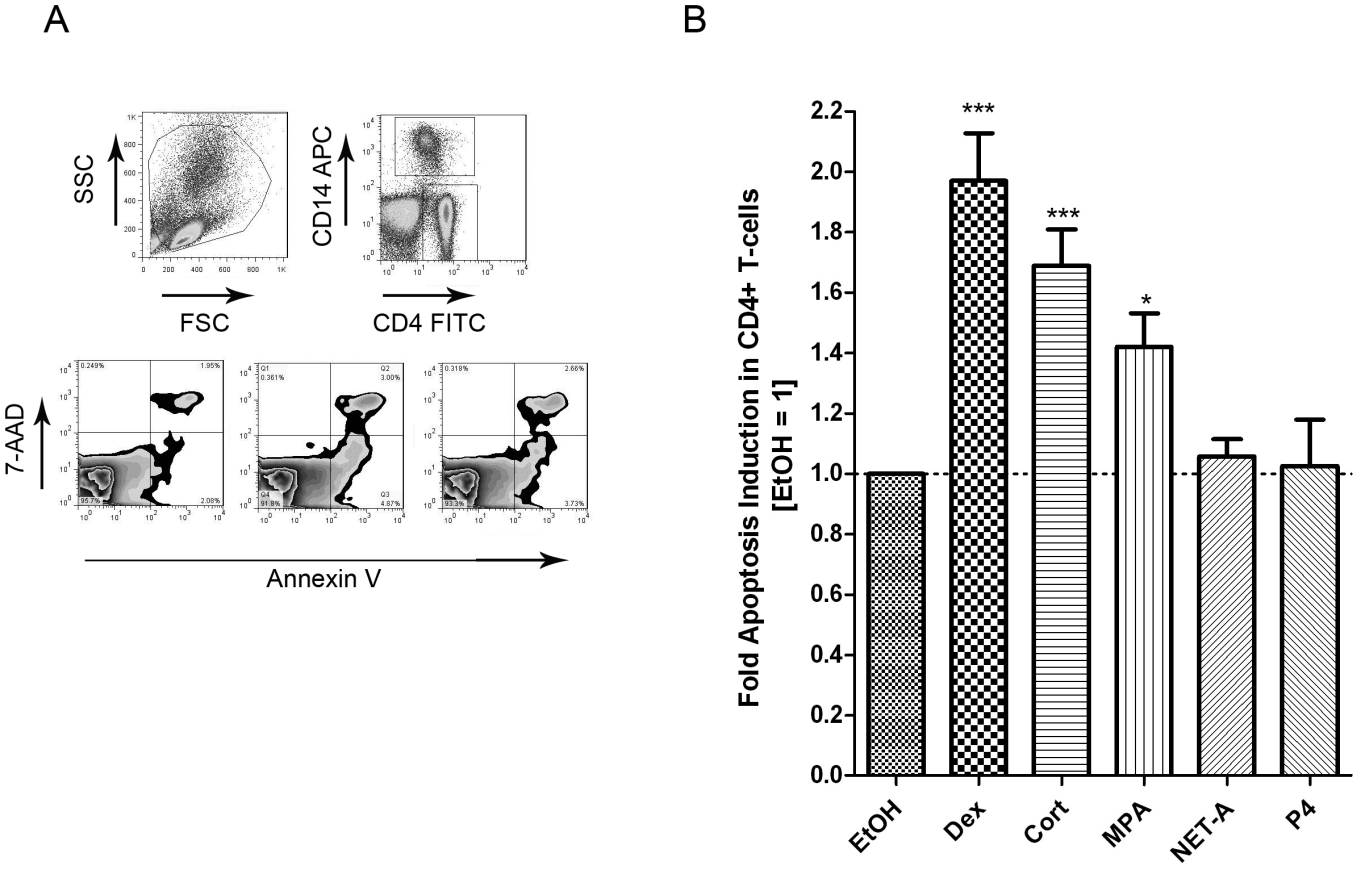
The progestin MPA, but not NET-A or P4, induces apoptosis in CD4^+^ T-cells. Cells were treated with or without 100 nM Dex, 100 nM F, 1 µM MPA, 10 µM NET-A, 1 µM P4 or vehicle control (EtOH) for 24 hrs. Cells were stained with anti-CD4, anti-CD14, annexin V and 7-AAD using the Apoptosis Detection kit I (BD biosciences). (A) Gating strategy and representative zebra plots of untreated (EtOH), MPA or Dex treated PBMCs. (B) The histogram shows pooled results from two independent experiments with samples from three donors. Data were acquired on a FACS calibur system (BD Biosciences) and analyzed using Flo-Jo software (Tree Star Inc., San Carlos, CA, USA). Statistical significance was determined by one-way ANOVA with Dunnett’s post-test, where *, **, and *** indicate p<0.05, 0.01 and 0.005 respectively. Error bars represent standard deviation.

GCs have been shown to induce apoptosis in several different cell lines, including CD4^+^ T-cells [35]. The progestin MPA is a partial to full GR agonist, unlike NET-A and P4 which have weak to no GR activity [30]–[33]. We investigated the relative capability of MPA and NET-A to induce apoptosis in CD4^+^ T-cells and CD14^+^ monocytes, as compared to the endogenous GC agonist F, the synthetic GR agonist Dex and P4. Briefly, PBMCs were isolated and treated with 100 nM Dex, 100 nM MPA, 10 µM NET-A, 1 µM P4 or vehicle control (EtOH) for 24 hrs. Cells were stained with anti-CD4 (T-cells), anti-CD14 (monocytes), 7-aminoactinomycin D (7-AAD), annexin V and the data were acquired using the Becton Dickinson FACS Calibur. 7-AAD was included to discriminate between live and dead cells. CD4^+^ T-cells and CD14^+^ monocytes were gated from the total PBMC population as indicated and the apoptotic cells were detected with the apoptosis marker annexin V (Figure 1A). As expected Dex and F induced apoptosis in a statistically significant manner in CD4^+^ T-cells by about 2-fold and 1.6-fold, respectively, compared to untreated cells (Figure 1B). Importantly, MPA also statistically significantly induced apoptosis in these cells (1.5-fold), yet to a lesser extent than Dex. By contrast, when cells were treated with NET-A or P4 no increase in apoptosis compared to control, was detected in CD4^+^ T-cells (Figure 1B). The apoptotic effect of Dex, F and MPA was however not observed in CD14^+^ monocytes (data not shown) and therefore the following experiments were carried out in CD4^+^ T-cells.

### MPA Acts Primarily via the GR to Induce Apoptosis in CD4^+^ T-cells

**Figure 2:**
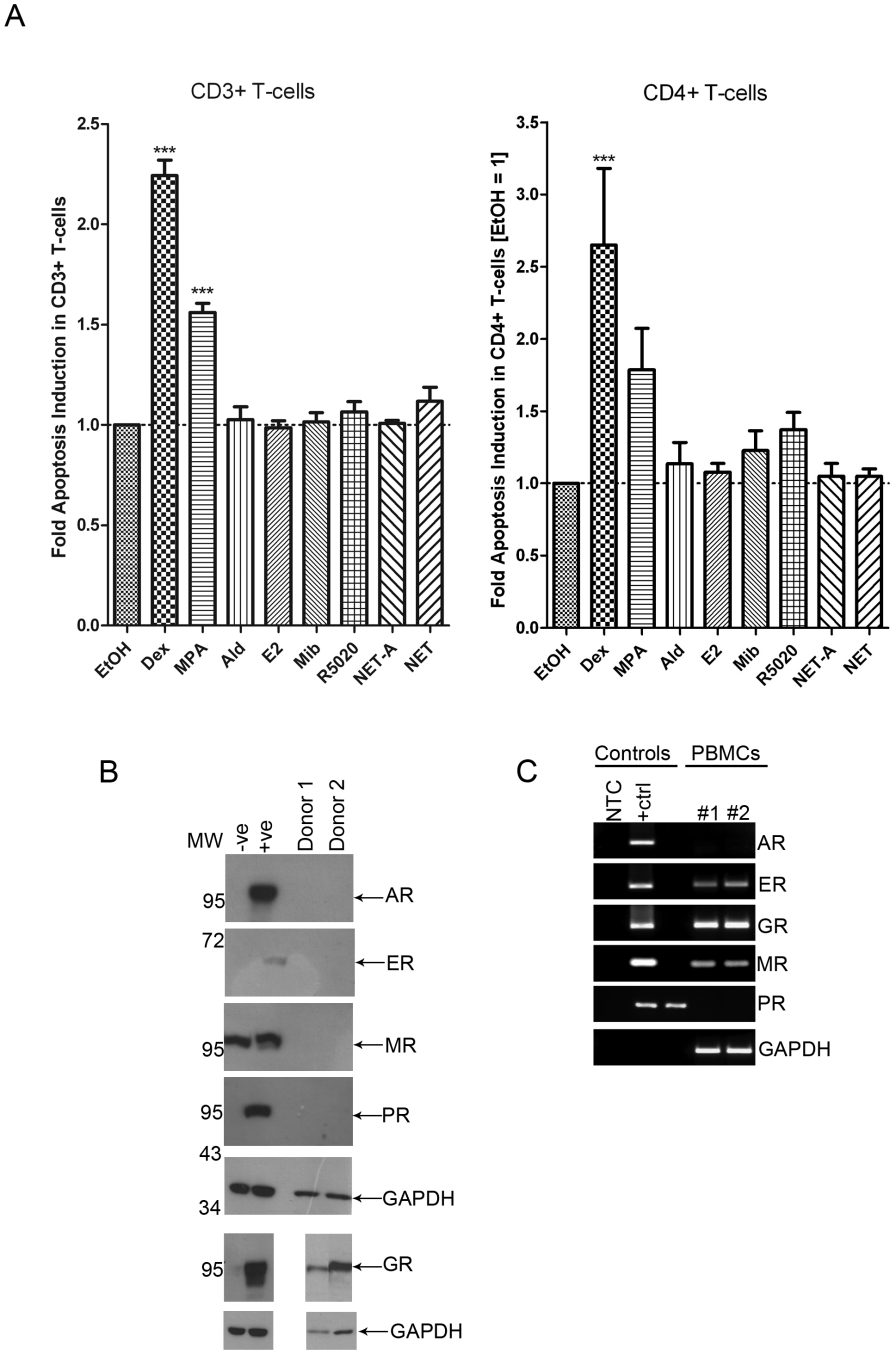
Apoptosis induction by Dex and MPA is most likely mediated primarily through the GR. (A) PBMCs were treated with vehicle (EtOH), 100 nM MPA, 10 nM Ald, 100 nM E2, 100 nM Mib, 100 nM R5020, 10 µM NET-A or 10 µM NET for 24 hrs at 37°C. Cells were surface stained with ant-CD3 and anti-CD4 antibodies, and apoptosis was detected using flow cytometry as described in Figure 1. The histogram shows pooled results from two independent experiments with samples from three donors. Statistical significance was determined by one-way ANOVA with Dunnett’s post-test, where *** indicates p<0.001. (B) Western analysis of lysates prepared from approximately 4×10^6^ PBMCs. Whole cell lysates of COS-1 cells overexpressing the relevant steroid receptor (+ve) or empty vector (−ve) served as the controls. GAPDH was used as a loading control. Note that the upper strong band on the MR blot is a COS-1 cell-derived non-specific signal which is absent for PBMCs, while the MR signal is the faint band just below the non-specific band which is only seen in the positive control. We were unable to obtain a more-specific anti-MR antibody. (C) Conventional PCR of cDNA prepared from human PBMCs using primers specific for the relevant steroid receptor. The controls were prepared by PCR amplification of the relative steroid receptor cDNA from plasmid DNA. GAPDH served as a control for mRNA levels. MW: molecular weight; NTC: no template control. Error bars represent standard deviation.

Next we sought to provide evidence that the increase in apoptosis observed with Dex and MPA was mediated via the GR and did not involve other steroid receptors. Since MPA is a PR agonist, and a partial agonist for the androgen receptor (AR) and a partial to full agonist for the GR [29], the possibility that MPA exerts its apoptotic effects via the PR or AR was investigated indirectly by using receptor-selective agonists. In order to determine whether other steroid receptors (apart from the GR) could induce apoptosis, PBMCs were treated with agonists that are selective for the AR (100 nM Mib), estrogen receptor (ER) (100 nM E2), mineralocorticoid receptor (MR) (10 nM Ald) and PR (100 nM R5020), as well as 100 nM Dex, 100 nM MPA, 10 µM NET-A or 10 µM NET for 24 hrs, and apoptosis was detected using flow cytometry as described previously. The ligands were used at saturating concentrations for each steroid receptor to control for the differences in relative binding affinities of each ligand for their respective receptors [33], [34], [94]. As found earlier, Dex significantly induced apoptosis by about 2-fold and about 3-fold in CD3^+^ and CD4^+^ T-cells, respectively (Figure 2A). MPA significantly induced apoptosis by about 1.5-fold compared to untreated control cells in the CD3^+^ T-cells and appeared to increase apoptosis in CD4^+^ T-cells to a similar extent as observed before (Figure 1B). In both CD3^+^ and CD4^+^ T-cells, the other steroid receptor-selective agonists did not induce apoptosis in a statistically significant manner (Figure 2A). Therefore, it is likely that the effects of MPA on apoptosis are mediated via the GR in T-cells. In support of these findings, PBMCs expressed GR protein whereas AR, PR, MR or ER protein expression was not detected by Western blot analysis (Figure 2B). The ER and MR mRNAs, but not AR or PR mRNAs, were however detected by PCR, indicating that ER and MR proteins may be expressed, but at a level undetectable by Western blot analysis (Figure 2C). Together with the results presented in Figure 2A, these data show that if low levels of ER and MR protein are expressed, they have no effect on apoptosis in CD3^+^ or CD4^+^ T-cells (Figure 2A). Taken together, these results strongly support the finding that the PR, AR, MR and ER do not induce apoptosis in these cells, and that MPA acts primarily via the GR to induce apoptosis in PBMCs. It is noteworthy that NET was included in this experiment as a control to exclude the possibility that the acetate form (NET-A) may be less active. However, similarly to NET-A, NET does not result in apoptosis.

### Dex and MPA but not NET-A or P4 Increase Apoptosis in a Dose-dependent Manner

**Figure 3:**
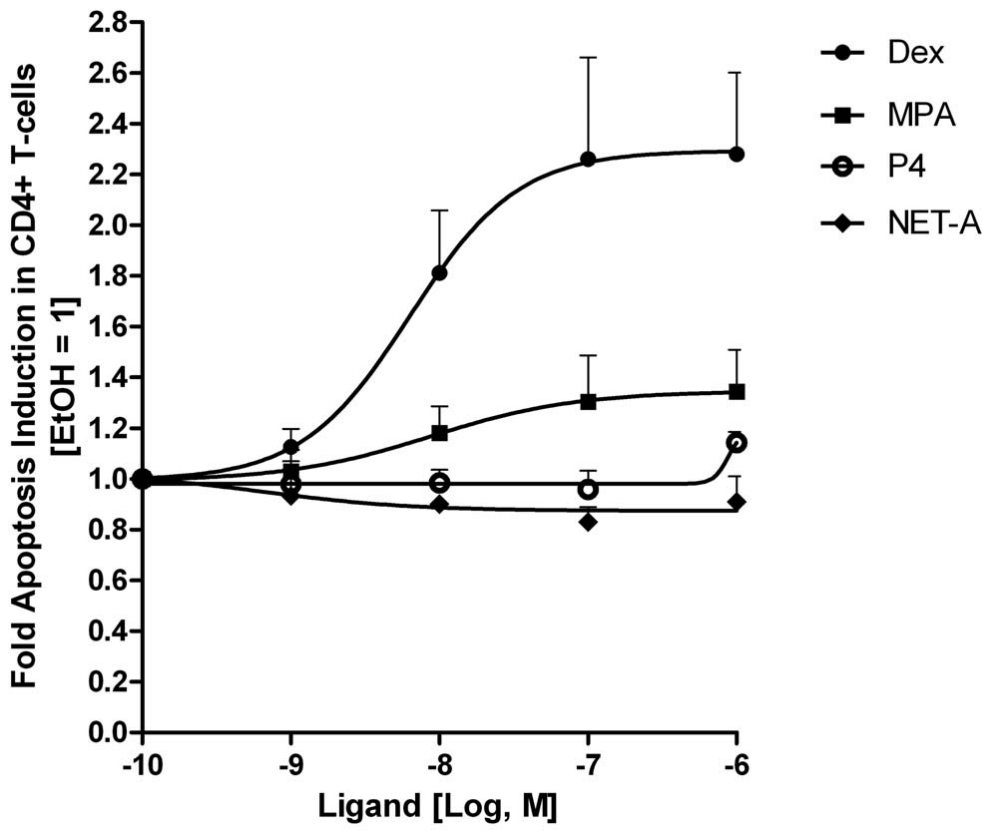
Dose-dependent apoptosis induction with Dex, MPA, NET-A and P4 in CD4^+^ T-cells. PBMCs were treated with vehicle (EtOH), Dex, MPA, NET-A or P4 at the concentrations indicated for 24 hrs at 37°C. Cells were stained and analysed as described for Figure 1. The figure shows pooled results from two independent experiments with samples from three donors. Error bars represent standard deviation. Statistical trend analysis for each dose response was performed by the Wilcox rank-sum test, as further extended by Cuzick [85], and showed a significant trend only for Dex (p<0.001) and MPA (p = 0.047).

Having established that both MPA and Dex induce apoptosis in CD4^+^ T-cells, we next sought to determine whether this pro-apoptotic effect was dose-dependent. Statistically significant trends [85] were observed for Dex (p<0.001) and MPA (p = 0.047), but not for P4 or NET-A, showing increased apoptosis with increasing concentrations of ligand, in the absence of Vpr, under these experimental conditions (Figure 3). Furthermore, apoptotic induction in response to both Dex and MPA was observed, starting at concentrations as low as 10 nM (Figure 3). The maximal apoptotic response observed for both Dex and MPA was reached at 100 nM, and the maximal response for MPA (∼1.3-fold) at that concentration was lower than for Dex (∼2.3-fold) (Figure 3). Note that the fold induction of apoptosis with MPA under these conditions varies between experiments from about 1.3- to 1.7-fold (Figures 1,2,3), most likely due to biological variability between donors. Even though no dose-dependent significant trend for changes in apoptosis was observed for NET-A or P4, a small response (∼1.1-fold) appeared to occur for P4 at 1 µM. These results are similar to dose-responses observed with these ligands for transcriptional regulation via the GR, with Dex acting as a full agonist and MPA as a partial agonist for the GR at concentrations between 1–100 nM, but with NET-A showing no agonist activity and P4 very weak to partial agonist activity in some contexts only at micromolar concentrations [31], [34]. Having established that both Dex and MPA induce apoptosis in the CD4^+^ T-cells in a dose-dependent manner, we next sought to determine if Dex and MPA can enhance Vpr-mediated apoptosis.

### Dex Enhances Vpr-mediated Apoptosis in a GR-dependent Manner

**Figure 4:**
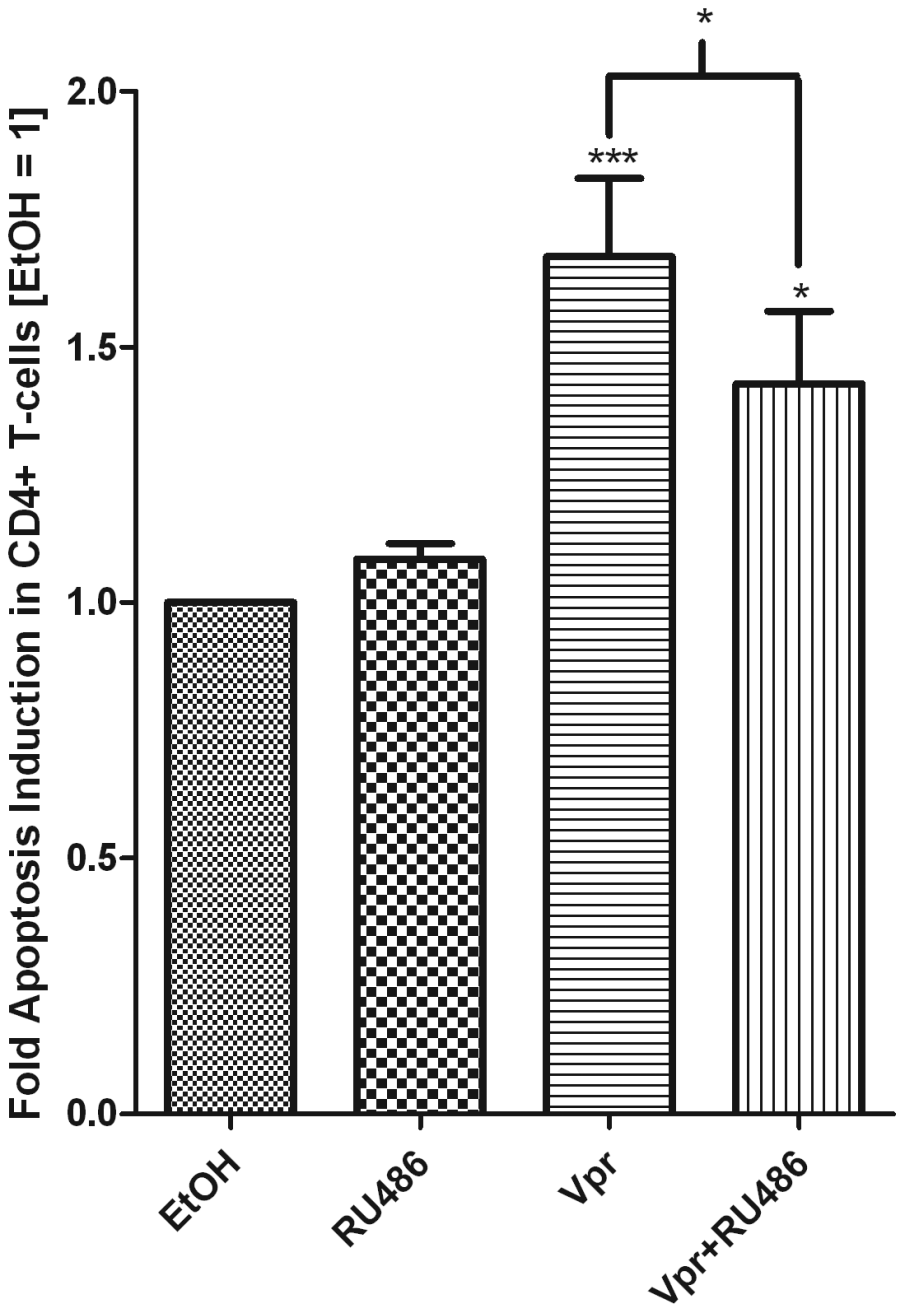
The GR is involved in Vpr-mediated apoptosis in CD4^+^ T-cells. PBMCs were treated with 1 µM RU486 in the absence or presence of 5 µM Vpr peptide (amino acids 52–96) for 24 hrs. A tryptic BSA digest served as a control (bars 1 and 2) wherever Vpr peptide was not added and was added at an equivalent mass/volume of peptide. Cells were obtained and stained as described in the methods. The histogram shows pooled results from two independent experiments with samples from three donors. Statistical significance was determined by one-way ANOVA with Dunnett’s post-test or a paired t-test, where *, **, and *** indicate p<0.05, 0.01 and 0.005 respectively. Error bars represent standard deviation.

**Figure 5:**
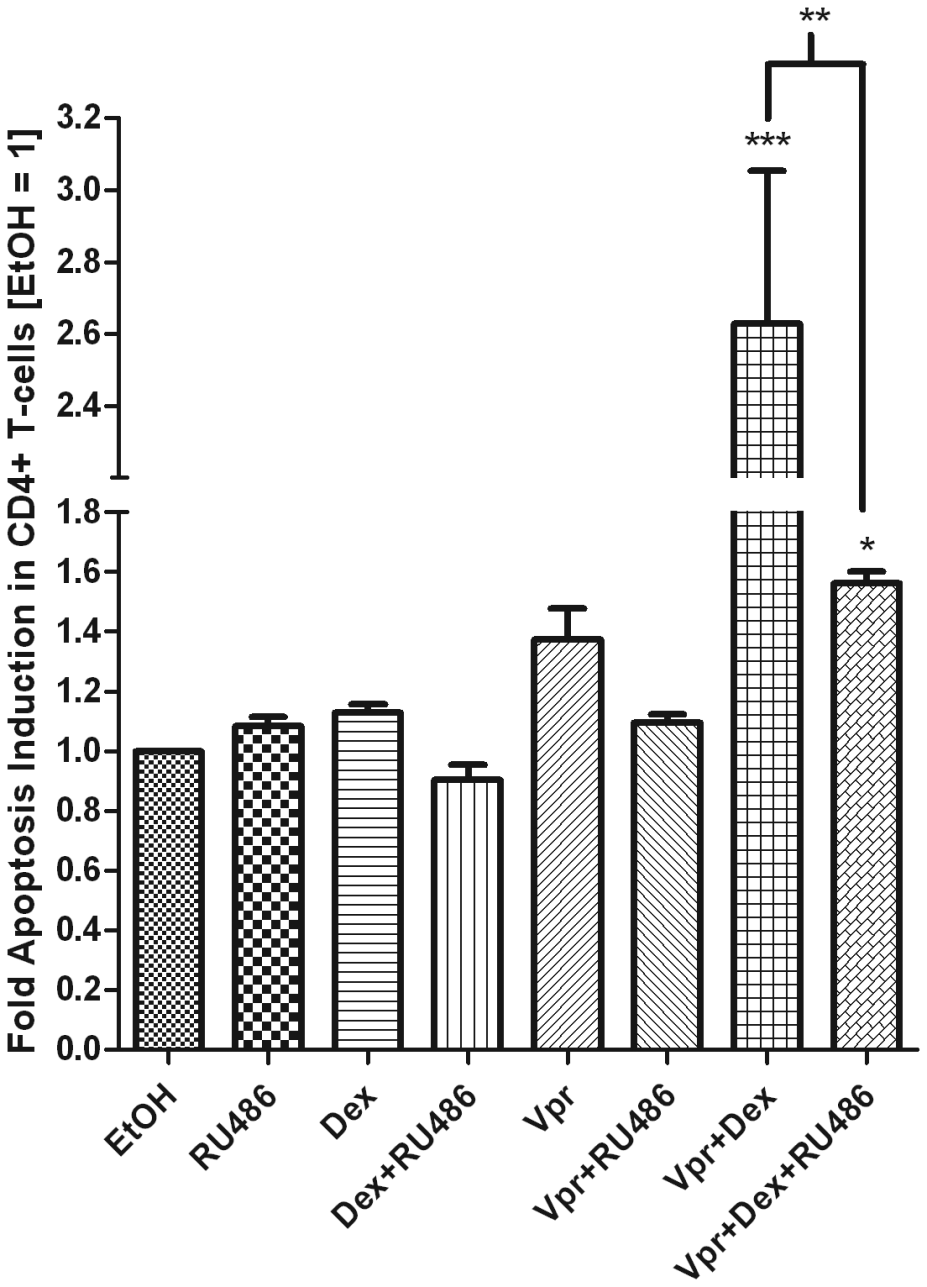
The GR is involved in GC- and Vpr-mediated apoptosis in CD4^+^ T-cells. PBMCs were treated with 100 nM Dex or 1 µM RU486 in the absence or presence of 5 µM Vpr peptide (amino acids 52–96) for 24 hrs. A tryptic BSA digest added at an equivalent mass/volume ratio of peptide, served as a control wherever Vpr peptide was present. Cells were obtained and stained as described in the methods. The histogram shows pooled results from two independent experiments with samples from three donors. Statistical significance was determined by one-way ANOVA with Dunnett’s post-test or a paired t-test, where *, **, and *** indicate p<0.05, 0.01 and 0.005 respectively.

It is well established that Vpr is a potent inducer of apoptosis in a number of different cell lines and primary cells. Therefore, we determined whether exogenous C-terminal Vpr peptide could induce apoptosis via the GR in CD4^+^ T-cells. As expected, Vpr peptide significantly induced apoptosis by approximately 1.8-fold in the CD4^+^ T-cells (Figure 4). This apoptotic induction was decreased in the presence of RU486, a potent GR antagonist, indicating that the GR was involved in Vpr-mediated apoptosis (Figure 4). We next determined whether Dex could enhance Vpr-mediated apoptosis through the GR in CD4^+^ T-cells. Cells were incubated with Vpr peptide in the absence and presence of Dex. Vpr and Dex alone induced apoptosis in CD4^+^ T-cells, although statistical significance could not be established, most likely due to the small responses (Figure 5). Furthermore, when cells were treated with Dex and Vpr in combination, a significant increase in apoptosis was observed as compared to Vpr or Dex alone. To establish whether the GR was involved in combined effects of Vpr- and Dex-mediated apoptosis, cells were treated in the absence and presence of RU486. RU486 alone had no effect on apoptosis (Figures 4 and 5). Apoptosis by Dex and Vpr alone was decreased in the presence of RU486, although statistical significance could not be established. Importantly, the 3-fold increase in apoptosis observed when cells were treated with Dex and Vpr in combination was significantly decreased in the presence of RU486 (Figure 5). Although RU486 is also a PR and MR antagonist [29], our data discount a role for these receptors in apoptosis in these cells (Figure 2). Taken together, the data suggest that the GR is required for Vpr- and Dex-mediated apoptosis, and suggests that the GR is required for Vpr enhancement of Dex-mediated apoptosis.

### MPA, but not NET-A or P4, Enhances Vpr-mediated Apoptosis in a GR-dependent Fashion

**Figure 6:**
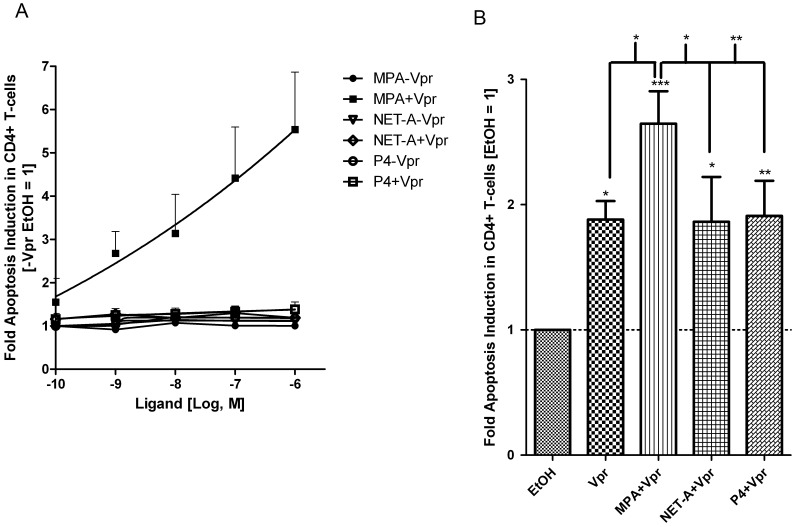
MPA but not NET-A or P4 increases Vpr-mediated apoptosis in a dose-dependent manner. (A) PBMCs were treated with or without 5 µM Vpr peptide (amino acids 52–96) and increasing concentrations of MPA, NET-A or P4 for 24 hrs. The graph shows results pooled from two independent experiments with samples from three donors. (B) PBMCs were treated with 100 nM MPA, 10 µM NET-A, 1 µM P4 or in combination with 5 µM Vpr peptide (amino acids 52–96) for 24 hrs. Cells were stained and acquired by flow cytometry as described in the materials and methods. A tryptic BSA digest served as a control wherever Vpr peptide was not added, as for results in figure 4. The histogram shows results pooled from two independent experiments with samples from three different donors compared to those in figure A. donors. Statistical trend analysis for panel A was performed by the Wilcox rank-sum test, as further extended by Cuzick [85], and showed a significant trend only for MPA plus Vpr (p = 0.012) and P4 minus Vpr (p = 0.012). Statistical significance in panel B was determined by one-way ANOVA with Dunnett’s post-test or a paired t-test, where *, **, and *** indicate p<0.05, 0.01 and 0.005 respectively. Error bars represent standard deviation.

Having shown that Dex treatment further increases Vpr-mediated apoptosis in a GR-dependent fashion, we next sought to investigate whether MPA, similarly to the full GR-agonist Dex, has the capability to enhance Vpr-mediated apoptosis. Under the experimental conditions used in figure 6, we observed statistically significant [85] trends only for MPA (p = 0.012) in the presence of Vpr, as well as for P4 in the absence of Vpr (p = 0.005), showing increased apoptosis with increasing concentrations of ligand (Figure 6A). Interestingly, a response was observed for MPA in the presence of Vpr at concentrations as low as 1 nM. A maximal increase of approximately 6-fold was obtained at a concentration of 1 µM MPA (Figure 6A). This was in contrast to cells treated in the absence of Vpr, where MPA appeared to have no dose-dependent effect on apoptosis (Figure 6A). The lack of apoptotic activity by MPA alone in these experiments compared to figures 1,2,3 was likely due to the conditions required to treat the cells with Vpr or control peptide, which masks the smaller effects of MPA alone. The dose response results are consistent with the results in Figure 6B, showing a statistically significant increase in Vpr-mediated apoptosis with MPA, but not NET-A or P4, using concentrations of ligands that nearly or fully saturate the GR.

**Figure 7:**
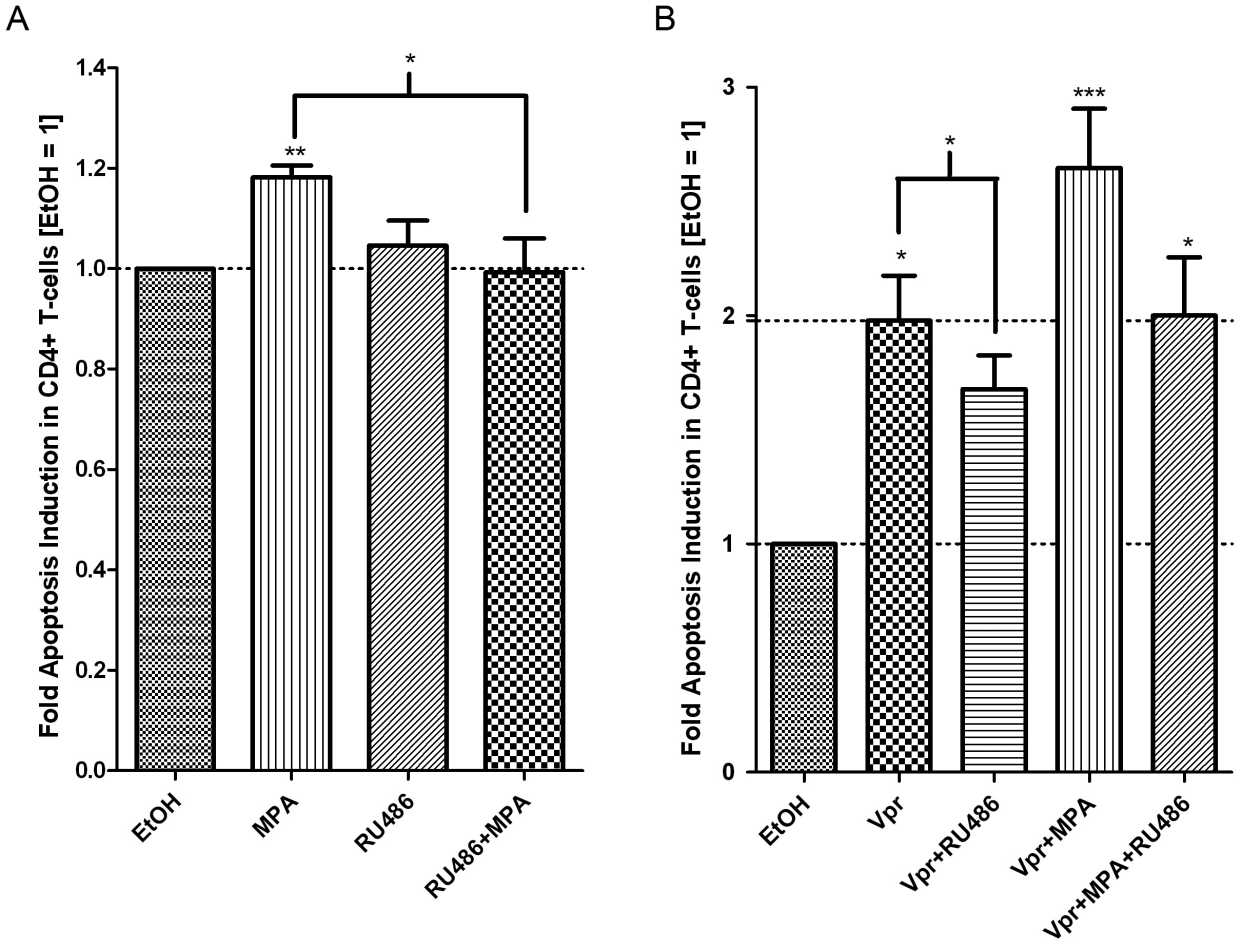
The GR is involved in MPA- and Vpr-mediated apoptosis in CD4^+^ T-cells. PBMCs were treated with vehicle (EtOH), 100 nM MPA, 1 µM RU486 or 100 nM MPA plus 1 µM RU486, in the absence (A) or presence (B) of 5 µM Vpr peptide for 24 hrs. Cells were stained and acquired by flow cytometry as indicated in the methods. In A cells were not incubated with balanced isotonic glucose-HEPES buffer while in B, this buffer was used and a tryptic BSA digest served as a control wherever Vpr peptide was not added, as described in Methods. The histograms show pooled results from two independent experiments with samples from three donors. Statistical significance was determined by one-way ANOVA with Dunnett’s post-test or a paired t-test, where *, **, and *** indicate p<0.05, 0.01 and 0.005 respectively. Error bars represent standard deviation.

Towards establishing a role for the GR in the MPA response, further experiments were performed with RU486, in the absence and presence of Vpr peptide (Figure 7). In the absence of prior incubation with peptide buffer (see methods), MPA significantly increased apoptosis compared to untreated CD4^+^ T-cells (Figure 7A), as previously shown (Figures 1,2,3). Importantly, although RU486 alone had no effect on apoptosis, this GR agonist could reverse MPA-mediated apoptosis in the CD4^+^ T-cells (Figure 7A) in a statistically significant manner. Vpr alone significantly induced apoptosis in CD4^+^ T-cells, and this response was decreased in the presence of RU486 (Figure 7A). Vpr and MPA in combination enhanced apoptosis by about 3-fold in a statistically significant manner, which was decreased by RU486 (Figure 7B). These results strongly suggest that MPA and Vpr alone or in combination, enhance apoptosis in CD4^+^ T-cells via a mechanism involving the GR. The lack of an effect by the natural PR ligand P4 or the synthetic progestin NET-A on Vpr-induced apoptosis is consistent with the requirement for GR agonist or strong partial agonist activity of a ligand to modulate Vpr-mediated apoptosis in CD4^+^ T-cells.

### Dex and MPA Enhance HIV-1-mediated Apoptosis in CD4^+^ T-cells

**Figure 8:**
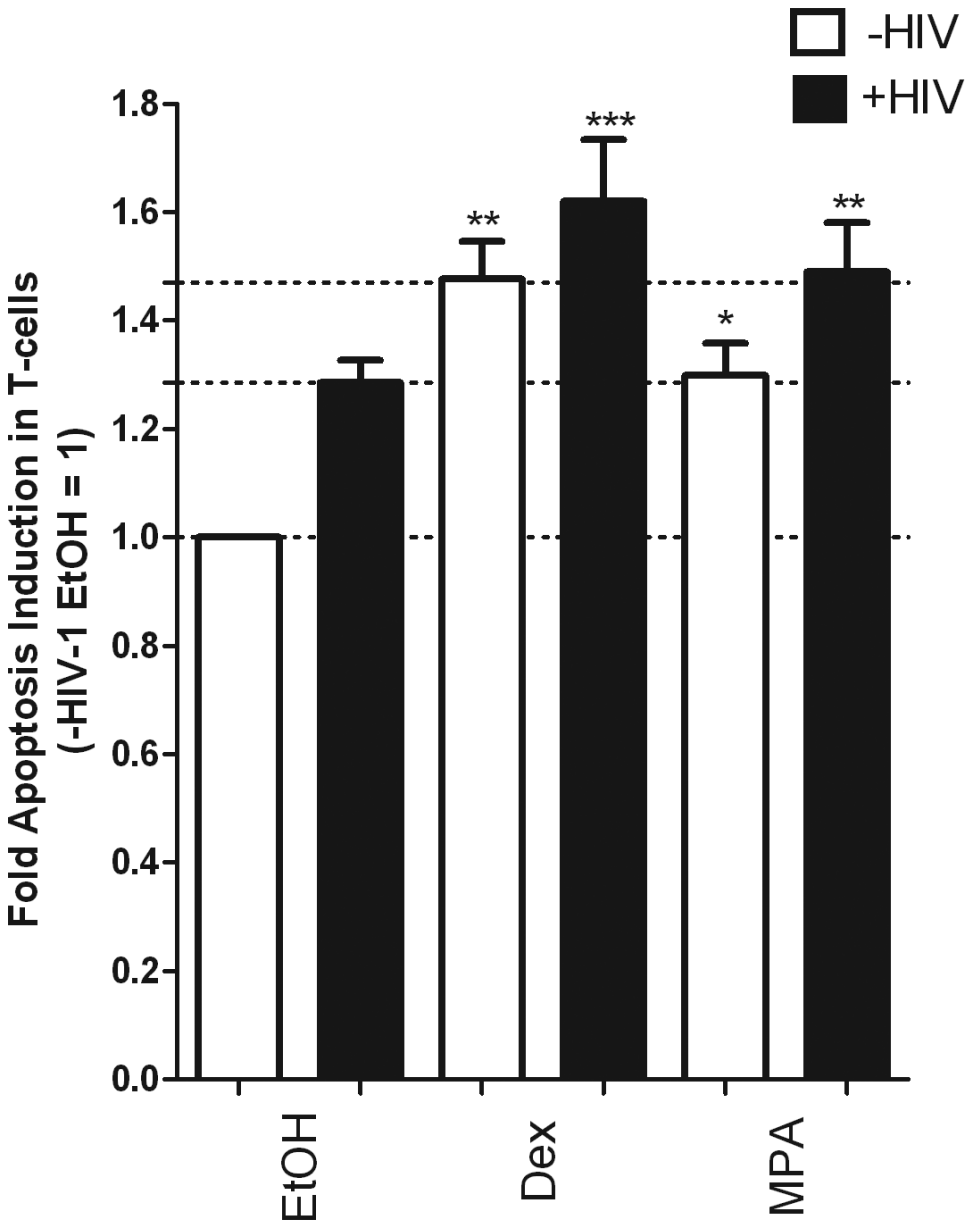
HIV-1-mediated apoptosis is enhanced in the presence of Dex and MPA. PBMCs were activated in the presence of PHA and rhIL-2 for 3 days at 37°C as described. Pseudotyped HIV-1 virus or control medium without virus was added to the cells, followed by incubation for a further 3 days to allow infection. Cells were then treated with vehicle (EtOH) or 100 nM Dex or MPA for an additional 24 hrs. Acquisition and analysis was carried out as described in the methods. The histogram shows pooled results from two independent experiments with samples from three donors. Statistical significance was determined by one-way ANOVA with Dunnett’s post-test, where *, **, and *** indicate p<0.05, 0.01 and 0.005 respectively. Error bars represent standard deviation.

Having shown that Dex and MPA enhance Vpr-mediated apoptosis using peptide studies, we next determined whether this effect could be elicited by intact HIV-1 pseudovirus. PBMCs were first activated with PHA and rhIL-2. Cells were then infected with pseudotyped HIV-1 virus for 3 days before being treated with the test compounds as indicated for an additional 24 hrs. The apoptotic phenotype was detected by flow cytometry as described above. However, we could not detect CD4^+^ T-cellsin this assay, which was most likely owing to decreased expression of the CD4^+^ receptor following T-cell activation and subsequent infection [96]. Thus, the results are representative of the T-cell population that was gated from the forward and side scatter plot. The responses observed from this PBMC population most likely represent the T-cell population only, because monocytes (which would scatter with the T-cells) are resistant to ligand- and Vpr-mediated apoptosis (data not shown; [97]). Consistent with results obtained in figures 1,2,3 and 5,6,7, stimulation with Dex and MPA resulted in a statistically significant increase in apoptosis (Figure 8). HIV-1 infection also increased apoptosis, which is consistent with results obtained with Vpr peptide (Figures 4,5,6,7) and in the literature [98]–[100]. Importantly, Dex and MPA stimulation further enhanced HIV-1 mediated apoptosis. In summary the data presented here indicate that Dex and MPA have the ability to increase T-cell apoptosis in the presence of HIV-1.

### Dex and Vpr Differentially Regulate Pro- and Anti-apoptotic Genes

**Figure 9:**
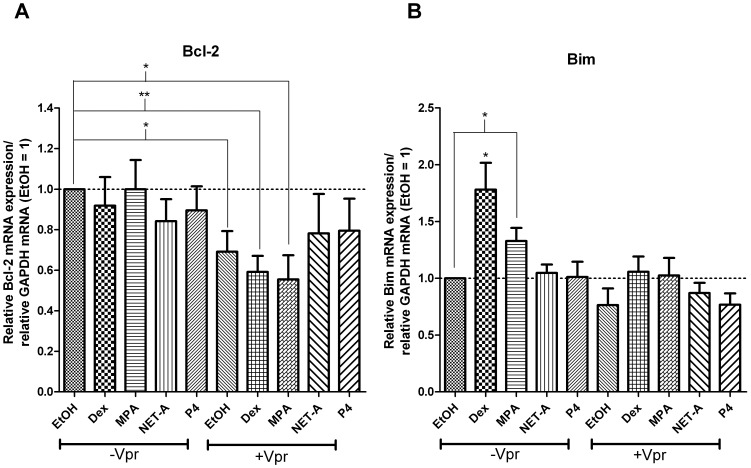
GC and Vpr differentially regulate key genes involved in apoptosis. PBMCs were treated with or without 5 µM Vpr peptide as described previously and treated with or without 100 nM Dex, MPA, NET-A or P4 for 24 hrs. After treatment, RNA was extracted, reverse transcribed, and Bcl-2 (A) or, Bim (B) mRNA expression was measured by real time PCR, normalising to GAPDH expression levels. The histogram shows pooled results from two independent experiments with samples from three donors. Statistical significance was determined by one-way ANOVA with Dunnett’s post-test, where *, **, and *** indicate p<0.05, 0.01 and 0.005 respectively. Error bars represent standard deviation.

The mechanism of apoptotic induction by the GR and Vpr in the presence of GR ligands most likely involves the transcriptional regulation of pro- and anti-apoptotic genes [35], [53]. To this end we set out to identify key genes that could be regulated by both Vpr and the GR. PBMCs were treated with or without 5 µM Vpr peptide in the presence or absence of 100 nM Dex, MPA, NET-A or P4 for 24 hrs (Figure 9 A and B). mRNA levels of potential target genes were determined by using real time PCR with specific primers to Bcl-2 or Bim. In the presence of Vpr alone or Vpr in combination with Dex or MPA, mRNA expression of the anti-apoptotic factor Bcl-2 was significantly repressed compared to vehicle-treated cells, although Dex, MPA, NET-A or P4 had no significant effect in the absence of Vpr (Figure 9A). However, Dex and MPA alone significantly increased the expression of the pro-apoptotic factor Bim by approximately 1.7-fold and 1.3-fold, respectively, whereas Vpr alone had no significant effect (Figure 9B). Both NET-A and P4 alone or in combination with Vpr peptide had no effect on Bcl-2 or Bim mRNA levels (Figure 9A and B). Additionally we found that Vpr and Dex alone or in combination had no effect on the pro-apoptotic genes Bcl-2-associated death promoter protein (Bad) and phorbol-12-myristate-13-acetate-induced protein 1 (NOXA) (data not shown). Taken together the data suggest that Vpr regulates different genes involved in the apoptotic pathway as compared to Dex and MPA, with Dex/MPA up-regulating Bim gene expression and Vpr decreasing Bcl-2 gene expression. Furthermore, the other steroid receptor-selective agonists (Ald, E2, Mib and R5020) did not affect expression of the genes investigated, indicating that the responses were most likely mediated by the GR (data not shown).

## Discussion

In this study we investigated the effects and molecular mechanisms of the injectable progestin contraceptives, MPA and NET-A, in CD4^+^ T-cells on apoptosis, in the absence and presence of the HIV-1 protein Vpr. It has previously been shown that GCs and Vpr induce apoptosis in T-cells [35], [53], that Vpr modulates GR function [70] and that MPA, but not NET-A, acts as a partial agonist for the GR [33], [34], [95]. We thus hypothesized that, similarly to GCs, MPA but not NET-A may also increase apoptosis in CD4^+^ T-cells, which may be further enhanced in the presence of Vpr. Consistent with this hypothesis, we show that the GR agonists Dex and F, as well as MPA, but not NET-A or P4, induce apoptosis in CD4^+^ T-cells. The predicted GR ligand response profile, and the inability of other steroid receptor-selective agonists to induce apoptosis in these cells, strongly suggests that the GR is the predominant receptor eliciting this effect. The relative responses by progestins are consistent with a lack of involvement of the PR, since MPA, P4 and NET are all potent PR agonists and hence apoptotic effects via the PR would be expected to be similar for these ligands, contrary to what is observed. On the other hand, MPA and NET-A have similar partial agonist activity via the AR and thus AR-mediated apoptotic effects via these ligands would be expected to be similar. Further support for the role of the GR is the finding that only ER, MR and GR mRNAs were detected in the PBMCs and only the GR protein but no other steroid receptor proteins were detected by Western blot analysis. MPA does not bind to and has no activity via the ER [29], while it binds very weakly but has no agonist activity on endogenous genes via the MR [101] and hence the progestins are unlikely to exert any apoptotic effects via these steroid receptors in PBMCs. Interestingly the AR, ER and MR have been shown to inhibit apoptosis in skeletal cells, breast cancer cells, neuronal cells and/or cardiomyocytes, when activated by their receptor-selective agonists, an effect that is most likely cell-specific [102], [103]–[105]. Our results showing no detectable PR or AR, but ER, MR and GR expression in PBMCs are consistent with the literature [49], [106], [107]. Furthermore, MPA-induced apoptosis could be inhibited in the presence of the GR antagonist RU486. A role for the GR in mediating apoptosis by MPA and not NET-A or P4 is consistent with the relative binding affinities, potencies (concentration for half maximal response) and efficacies (maximal response) for transcriptional regulation by these ligands via the GR [31]–[34], [95].

As expected, Vpr alone induced apoptosis in CD4^+^ T-cells, which was further increased dose-dependently in the presence of Dex or MPA. Remarkably, MPA appeared to enhance Vpr-mediated apoptosis at a concentration as low as 1 nM (Figure 6A) which is lower than the peak and plateau levels observed in the serum of female patients using DMPA [87]. These findings suggest that the presence of MPA during HIV-1 infection would further potentiate the effects of Vpr on apoptosis in T-cells. As hypothesized due to their weak GR activity, NET-A or P4 did not induce apoptosis alone or in combination with Vpr. The increase in apoptosis observed with MPA, Dex or Vpr alone or Dex and MPA in combination with Vpr was decreased by the GR antagonist RU486, indicating that the GR is required for these effects. This is the first report to our knowledge showing that the GR is required for Vpr-mediated apoptosis in primary T-cells. The physiological significance of these findings with Vpr require further investigation. From the literature, it is unclear at what concentration Vpr occurs in the serum of infected individuals. One report has detected Vpr in the serum of infected patients at the same concentrations as circulating viremia [56], whereas another suggests that Vpr is present at a concentration of 0.7 nM [108]. Higher concentrations of Vpr peptide as used in this study (5 µM) and by others (1–10 µM) are required to induce apoptosis *in vitro* [61], [62], [99], [109]. The intracellular concentrations of Vpr protein delivered and/or expressed in specific T-cells during chronic infection are unknown and likely to be much higher than serum concentrations reflecting Vpr diluted in the total volume of blood in the body. Thus whether the concentrations of Vpr peptide used in this study are physiologically relevant is not possible to ascertain at present. Interestingly, it has been reported that low concentrations of Vpr protect T-cells from apoptosis [110]. These authors suggested that the levels of Vpr during infection may vary in a manner that may be crucial to maintaining viral virulence and increased pathogenesis. Thus, it is possible that the levels of Vpr vary both in specific cellular environments and during different stages of disease progression such that at low levels of Vpr, apoptosis of T-cells does not occur to favour viral replication, whereas at other stages of the disease, increased Vpr levels may favour apoptosis and T-cell death.

To investigate whether the results with Vpr peptide are consistent with a role for Vpr delivered in the context of the whole virus we treated PBMCs with or without HIV-1 pseudotyped virus in the absence and presence of MPA and Dex. The results showed that MPA and Dex increase apoptosis induced by the HIV-1 pseudovirus particles. This result is consistent with potentiation by GR ligands of apoptosis in the presence of HIV-1 proteins. However, it does not exclude the possibility that other proteins besides Vpr are involved in the response in the context of HIV-1 pseudovirus particles.

Even though the literature suggests that Vpr directly targets the mitochondria during apoptosis, there is evidence that Vpr is predominately localized to the nucleus [66], [111]–[113]. It is possible that a small percentage of Vpr translocates to mitochondria, but requires the transcription of pro-apoptotic genes in the nucleus to fully commit to apoptosis. For this reason, Vpr may regulate host gene expression to induce apoptosis. To determine which genes are involved in Vpr-mediated apoptosis in the presence of GR ligands we investigated key genes that have previously been shown to be regulated by either GCs or Vpr. The anti-apoptotic factor Bcl-2 was previously identified as a key mediator of apoptosis because its overexpression in a murine lymphoma cell line protected cells from GC-induced apoptosis [114]. Bcl-2 has been shown to be down-regulated by Vpr in a human promonocytic cell line [97], [109]. Key genes that are upregulated by GCs include Bim in human and murine leukaemia cell lines as well as primary murine thymocytes [115]. As shown previously [109], the Vpr peptide down-regulated the anti-apoptotic gene Bcl-2. In contrast, we show that Dex and MPA, but not NET-A or P4, increased the expression of the pro-apoptotic genes Bim. Both Vpr and Dex alone or in combination had no effect on the pro-apoptotic genes NOXA and Bad (data not shown). Furthermore, no other steroid receptor-selective agonist enhanced or decreased expression of Bim or Bcl-2, indicating that the GR was the only steroid receptor that increased Bim expression (data not shown). Surprisingly Vpr and Dex did not act in concert to regulate gene expression of any genes tested. The evidence presented here suggests that the GR and Vpr differentially regulate either pro- or anti-apoptotic genes, most likely resulting in a potent apoptotic response over a prolonged period of time. In the absence of Vpr, apoptosis is favoured by GCs or the progestin MPA, by induction of the pro-apoptotic gene Bim, whereas in the absence of GCs or MPA but the presence of Vpr, apoptosis is favoured by the repression of the anti-apoptotic gene Bcl-2. It is likely that the differential regulation of apoptotic genes by GCs/MPA and Vpr contributes to increased pathogenicity of the virus and T-cell depletion. We cannot however rule out the possibility that GCs/MPA and/or Vpr regulate other genes involved in the apoptosis pathway, or that Vpr induces apoptosis through direct interaction with the mitochondrial membrane or that the extrinsic and intrinsic pathways act together in inducing apoptosis in the CD4^+^ T-cells.

Taken together, these findings are consistent with a role for MPA in repressing systemic immune function by increasing apoptosis in CD4^+^ T-cells in the absence of HIV-1 infection, and an increase in this effect in the presence of HIV-1 infection. Furthermore the findings suggest that this occurs via a mechanism involving the GC-like properties of MPA, via GR-mediated changes in transcription of apoptotic genes, which are involved in the intrinsic apoptotic pathway. The extent to which these systemic immunosuppressive effects are physiologically relevant requires further investigation in clinical models, but the dose response results suggest that the apoptotic effects of MPA could occur within the peak nanomolar physiological concentration range measured in serum samples of women on DMPA. Additionally, the results in Figure 6 suggest that even when the MPA concentrations drop to about 2.6 nM a few weeks after injection of DMPA, MPA could potentiate apoptotic effects of Vpr in HIV-1 infected patients during chronic infection. The apoptotic effects of MPA in the absence of HIV-1 infection could have a role in acquisition of the virus owing to compromised immune responses, whereas the effects of DMPA in the presence of HIV-1 could have a role in disease progression and depletion of the T-cell population. These results for MPA are consistent with results showing a slower cellular immune response rate in DMPA-treated animals [40] and decreased T-cell numbers in patients treated with high concentrations of MPA for breast cancer [41].

There is not much information available regarding the effects of DMPA contraceptive usage on T-cell populations in women. Synthetic hormones in combined oral preparations, which usually do not contain MPA, were found not to affect absolute numbers or percentages of lymphocytes, T-cells and subsets of T-cells [49], [116], consistent with a lack of an effect of contraceptives other than MPA on T-cell apoptosis. However, our results are consistent with the findings showing accelerated loss of CD4^+^ T cells and death rate in women on DMPA infected with HIV-1 compared to non-contraceptive users [12]. The results in this study are also consistent with results for women using MPA in HRT, who exhibited a decrease in total lymphocyte count [117], the percentage of T-cells [117], [118] and the percentage of T-helper (Th) lymphocytes [117].

Our finding that NET-A does not exhibit these GR-mediated apoptotic effects like MPA and F, suggests that choice of progestin contraceptive could significantly affect susceptibility to and progression of infectious diseases, such as HIV-1 and AIDS. The finding that P4 at concentrations less than 1 µM does not induce apoptosis in T-cells suggests that P4 concentrations in the luteal phase of the menstrual cycle [29] are unlikely to affect immune function via apoptosis of T-cells. These findings highlight the fact that not all progestins are the same [29], [31], [33], [119] and that choice of progestin in hormonal therapy needs to be carefully considered. The choice of progestin for contraception may be particularly important for young women of child bearing age in the developing world in high risk areas for HIV-1 infection, where MPA usage as an injectable contraceptive is high [120].
